# Audiovisual quality impacts assessments of job candidates in video interviews: Evidence for an AV quality bias

**DOI:** 10.1186/s41235-018-0139-y

**Published:** 2018-12-07

**Authors:** Joshua L. Fiechter, Caitlan Fealing, Rachel Gerrard, Nate Kornell

**Affiliations:** Department of Psychology, Williams College, 25 Stetson Ct., Williamstown, MA 01267 USA

## Abstract

Video job interviews have become a common hiring practice, allowing employers to save money and recruit from a wider applicant pool. But differences in job candidates’ internet connections mean that some interviews will have higher audiovisual (AV) quality than others. We hypothesized that interviewers would be impacted by AV quality when they rated job candidates. In two experiments, participants viewed two-minute long simulated Skype interviews that were either unedited (fluent videos) or edited to mimic the effects of a poor internet connection (disfluent videos). Participants in both experiments rated job candidates from fluent videos as more hirable, even after being explicitly told to disregard AV quality (experiment 2). Our findings suggest that video interviews may favor job candidates with better internet connections and that being aware of this bias does not make it go away.

## Significance statement

Employers are increasingly relying upon video-chat services such as Skype to conduct job interviews. Video interviews allow employers to assess a wider array of prospective employees and they incur less monetary and time costs than do in-person interviews. However, video interviews also introduce new concerns; specifically, employers’ assessments of candidates may be negatively influenced by the audiovisual (AV) quality of a video interview. In two experiments, we had people view short clips of simulated Skype interviews. Some of these clips were edited to mimic poor AV quality. People rated candidates from high-quality videos as more hirable, suggesting that AV quality does, in fact, influence hiring decisions. Furthermore, in our second experiment, we explicitly warned people not to allow AV quality to influence their assessments of the job candidates. Despite this warning, candidates from high-quality videos were still rated as more hirable. Overall, our findings suggest that job candidates with poor internet connections and/or slow computers are at a disadvantage in video interviews, and that this disadvantage persists even when interviewers are explicitly instructed to discount AV quality in hiring decisions.

Job interviews are frequently conducted on video-chat services such as Skype (Schoen, 2014). One problem with this development is that audiovisual (AV) quality can vary considerably across interviewees. We asked whether AV quality affects hiring decisions. If such an AV quality bias exists, then candidates with faster devices or internet connections might be hired more often than those without, even if they are not more qualified.

Past research shows that impression formation is affected by fluency, which we define as the subjective feeling of ease or difficulty one experiences when processing information. Fluent processing is associated with more positive ratings than disfluent processing across a wide variety of judgments, including aesthetic beauty of basic shapes (Reber, Winkielman, & Schwarz, 1998), truthfulness of written statements (Reber & Schwarz, 1999), instructor ratings (Carpenter, Wilford, Kornell, & Mullaney, 2013), and memorability of words (Rhodes & Castel, 2008), among others (see Alter & Oppenheimer, 2009, for a review). The assessments we make of other people are also affected by fluency (see Lick & Johnson, 2015, for a review). One especially relevant study found that, in computer-mediated conversation, introducing a brief lag in auditory and visual feedback caused participants to feel less solidarity with each other (Koudenburg, Postmes, & Gordijn, 2013).

Previous research on job interviews is consistent with the hypothesis that decreased fluency is associated with lower ratings. For example, interviewers assign lower ratings to job candidates who speak with an accent (Hosoda, Nguyen, & Stone-Romero, 2012; Hosoda & Stone-Romero, 2010) or have a facial stigma such as a scar (Madera & Hebl, 2012). However, no prior research has evaluated the effect of AV fluency on ratings of job candidates, and there are reasons to doubt that these variables are correlated. Unlike accent and appearance, AV fluency is not an attribute of the candidate him or herself. Furthermore, multiple studies have failed to replicate fluency effects, which suggests that they can be fickle (e.g., Geller, Still, Dark, & Carpenter, 2018; Meyer et al., 2015; Rummer, Schweppe, & Schwede, 2016).

In the present experiments, we manipulated processing fluency by simulating the effects of a bad Skype connection. Simulated Skype interviews were edited to be either fluent (high AV quality) or disfluent (decreased visual resolution, pauses in the video, and background noise). We predicted that job candidates whose interviews had lower AV quality would be rated as less hirable.

## Experiment 1

### Method

Our complete method for both experiments, including sampling plan and reported statistical analyses, was preregistered at the Open Science Framework (OSF; https://osf.io/h7u68/). We analyzed our data using Bayesian *t*-tests (Rouder, Speckman, Sun, Morey, & Iverson, 2009). One advantage of Bayesian analyses is the option to stop gathering data once a desired result has been obtained (Rouder, 2014; for a mathematical proof see Deng, Lu, & Chen, 2016). We therefore planned to collect data in increments of 40 people, stopping either when 1) the Bayes factors supported the null or alternative hypothesis by a magnitude of 3 or greater or 2) when we had collected data from 200 people.

#### Participants

We recruited 97 people from Amazon’s Mechanical Turk Service. We initially collected data from 120 people, and then excluded participants who 1) did not complete every phase of the experiment, 2) started the experiment multiple times, 3) reported experiencing technical problems, 4) did not indicate that they were fluent in English, or 5) reported seeing our stimuli before.

#### Design

We used a two-level (AV quality, fluent or disfluent) within-subject design.

#### Stimuli

Stimuli were four simulated video interviews, each featuring a different actor. All actors were filmed in the same location. The actors were a Caucasian female, an Indian male, an Asian female, and an African-American male. We made two versions of each video: a fluent version, which was kept at maximum AV quality, and a disfluent version, which was edited using Final Cut Pro X so that the visual and sound quality were degraded (these videos are also available at https://osf.io/h7u68/). Visual quality was manipulated by adding freeze frames to simulate picture freezing during the interview and by adding a light-balance distorting visual filter. Sound quality was manipulated with a high-pass audio filter with a cutoff frequency of 6900.0 and a resonance of 0. (In-video volume was increased to partially counteract the volume difference between the fluent and disfluent videos.) The audio feed never paused, so participants were able to hear every word spoken in the video, but there was background static noise. The durations of the videos were 105, 116, 156, and 173 s. Most actual interviews are not this brief, but impressions formed in a few seconds often match up closely with impressions formed over the course of hours (Ambady & Rosenthal, 1992). There was no difference in duration between the fluent and disfluent videos of the same actor.

### Procedure

Participants were told that they would be watching segments from four interviews for a legal secretary position and that they would rate the candidates once they had watched all the videos. They were not told that AV quality would vary between videos. The videos were presented in the same order for every participant. The fluency of the videos was randomly selected from one of two predetermined arrangements: 1) the first and last videos were disfluent or 2) the middle two videos were disfluent.

We tried to ensure that participants were paying attention in two ways. First, a button with the label “Press me now” would periodically appear onscreen as the videos played; participants were instructed to click this button as quickly as possible. Second, immediately following each video, participants were asked three basic questions about the candidate’s responses (e.g., “Where did the candidate say they attended college?”).

After all of the videos had been viewed, participants rated how hirable each candidate was on a scale from 1 (“I would never hire this person”) to 10 (“I would certainly hire this person”). The ratings were made in the same order that the interviews were seen. Participants then cycled through all candidates again, rating each candidate on likeability from 1 (not at all likeable) to 10 (extremely likeable).

### Results and discussion

As noted previously, we analyzed our data using Bayesian *t*-tests (Rouder et al., 2009). We will report Bayes factors in terms of support for the alternative hypothesis (BF_10_). A BF_10_ greater than 1 indicates support for the alternative and a value less than 1 indicates support for the null. We consider values greater than or equal to 3 (or less than or equal to 0.33) as offering convincing evidence for the alternative (or null) hypothesis. In our analyses, a BF_10_ ≥ 3 will always correspond to a *p* < 0.05.

**Fig. 1:**
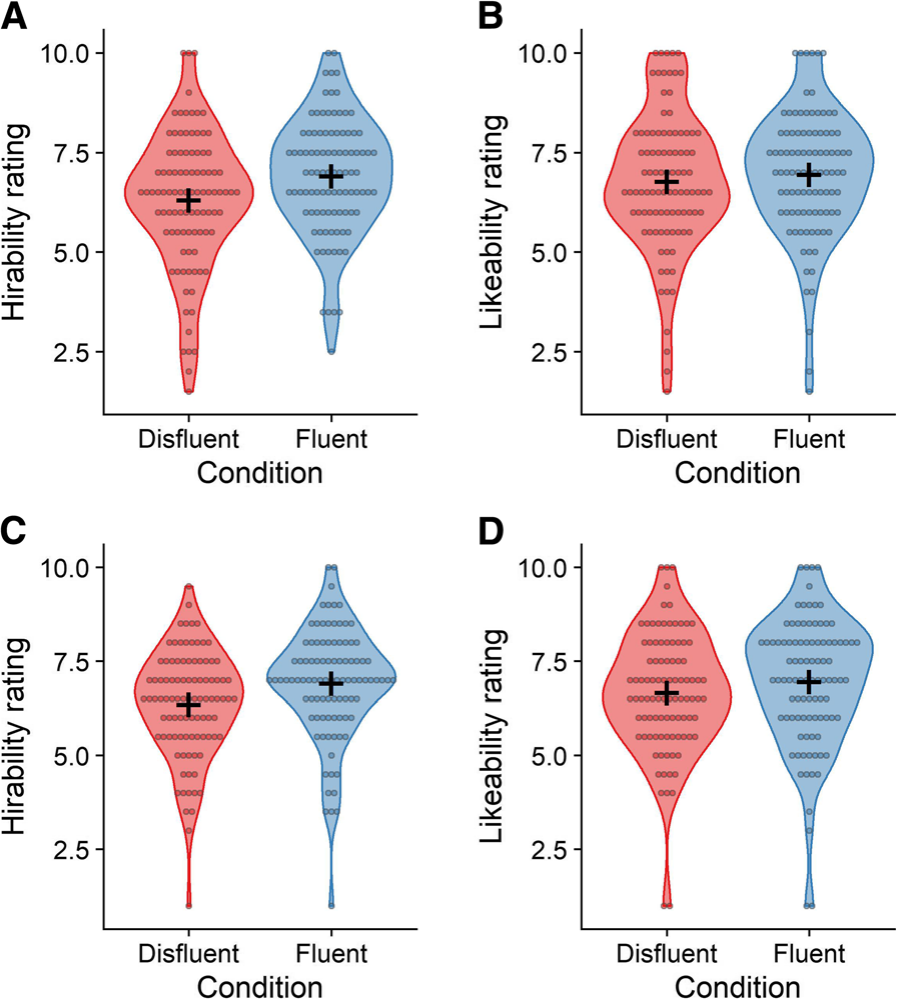
Hirability and likeability ratings as a function of AV fluency in experiments 1 (**a**, **b**) and 2 (**c**, **d**). *Circles* represent the mean rating for each participant. *Crossed lines* indicate condition means

Employability and likeability ratings in each condition are presented in Fig. 1a, b, respectively. Candidates in fluent videos were rated as more hirable (*M* = 6.91, *SD* = 1.46) than candidates from disfluent videos (*M* = 6.31, *SD* = 1.69), BF_10_ = 5.62, Cohen’s *d* = 0.42. Responses to the likability question for fluent videos (*M* = 6.95, *SD* = 1.60) compared to disfluent videos (*M* = 6.77, *SD* = 1.74) supported the null hypothesis, BF_10_ = 0.17. In short, experiment 1 demonstrated an AV quality bias: candidates from disfluent videos were rated as less hirable.

## Experiment 2

In experiment 2, we attempted to reduce the impact of fluency by warning our participants that they should not let AV quality influence their ratings. Making participants aware of the effects of fluency has been effective in reducing its influence in some previous studies (Lev-Ari & Keysar, 2010; Oppenheimer, 2006) but not others (Kelley & Lindsay, 1993; Rhodes & Castel, 2008).

### Method

#### Participants

We recruited 96 people from Amazon’s Mechanical Turk service. We initially collected data from 120 people and then excluded participants following the same rules as in experiment 1.

#### Design, stimuli, and procedure

The designs, stimuli, and procedures of experiments 1 and 2 were identical with one exception. Immediately prior to viewing the first interview, participants in experiment 2 received the following warning:

- Please read carefully: You will be watching videos that are of good and poor quality. Research has shown that the quality of video or audio can impact assessments of job candidates. As you watch the interviews, try not to let video quality bias you for or against any of the candidates.

### Results and discussion

Employability and likeability ratings in each condition are presented in Fig. 1c, d. The results replicated experiment 1: Candidates were rated as more hirable when AV quality was good (*M* = 6.91, *SD* = 1.48) than when it was poor (*M* = 6.35, *SD* = 1.42), BF_10_ = 15.78, *d* = 0.47. Likeability was, again, similar for candidates in the fluent (*M* = 6.96, *SD* = 1.71) and disfluent videos (*M* = 6.66, *SD* = 1.61), though unlike experiment 1, we did not find convincing evidence in support of the null hypothesis, BF_10_ = 0.65.^1^ Once again, participants preferred candidates from fluent videos, even after being explicitly warned about the biasing effect of AV quality.

## Omnibus analysis

Because our experiments were nearly identical in their methods, we combined the data from the two studies to assess the totality of our evidence. (These combined analyses were not preregistered.) Candidates from fluent videos were rated as more hirable (*M* = 6.91, *SD* = 1.47) than were candidates from disfluent videos (*M* = 6.33, *SD* = 1.56), BF_10_ = 524.51, *d* = 0.44. The likability of candidates in fluent videos (*M* = 6.96, *SD* = 1.65) and the disfluent videos (*M* = 6.72, *SD* = 1.67) were not significantly different, though our evidence did not conclusively favor the null hypothesis either, BF_10_ = 0.52.

In a final set of analyses, we assessed which candidate would be offered the job. To do so, we categorized each participant into one of three groups based on whether they gave their highest hirability rating to a fluent candidate, disfluent candidate, or both. The number and proportion of participants in each of these three categories is displayed in Table 1. We then analyzed only the ratings from those participants for whom we could infer a fluency preference (i.e., those in the top two rows of Table 1); we specifically wanted to know if fluent candidates received a majority of the highest ratings. Of the 162 participants who assigned their highest rating to a single condition, 104 (64%) favored a fluent candidate, BF_10_ = 110.86. Some job interviews—and particularly remote interviews—are conducted with the aim of weeding out those candidates who are least preferred. In consideration of this fact, we also analyzed the frequency with which participants assigned their lowest hirability rating to candidates from fluent and disfluent videos (Table 1). Of the 158 participants who assigned their lowest rating to a single condition, 99 (63%) least preferred a disfluent candidate, BF_10_ = 26.38.

**Table 1:** Number of participants (proportion in parentheses) across both experiments who assigned their highest and lowest hirability rating to a job candidate from a fluent video, disfluent video, or both (*N* = 193)

Condition	Highest rating	Lowest rating
Fluent	104 (0.54)	59 (0.31)
Disfluent	58 (0.30)	99 (0.51)
Both	31 (0.16)	35 (0.18)

## General discussion

Our results offer the first evidence that AV quality impacts decision making in job interviews. Job candidates were rated as more hirable when the AV quality of their interviews was better. We also found that warning participants that they should not allow AV quality to influence their ratings did not eliminate this effect.

Likeability ratings were not significantly impacted by AV quality. We hesitate to speculate too much about this finding because the data did not conclusively support the hypothesis that AV quality does not affect likability ratings. However, one possibility is that participants used likability as one of the features that guided their hirability ratings (which were always assessed first). Consequently, likeability ratings may have reflected only those components of likeability that had not already influenced hirability (Schwarz, 1999). Another possibility is that fluent processing does not affect likeability, as has been suggested by prior studies (Jakesch, Leder, & Forster, 2013).

Participants in experiment 2 failed to discount AV fluency. It is possible that fluency influenced them at an implicit level, they were not aware of it, and therefore did not adjust for it. There are other possible explanations as well. First, being asked to press a button at random timepoints while they viewed the videos may have divided participants’ attention, which might have made discounting fluency more difficult (Oppenheimer & Monin, 2009). Second, our participants might have failed to discount AV quality because they did not think doing so was appropriate, despite our instructions; for example, they might have believed that poor AV quality is reflective of an unprepared candidate (e.g., because the candidate failed to test their connection before the interview).

The AV quality bias has troubling implications for job interviews, especially because it might put people who have inferior devices or internet connections, such as rural or poor people, at a disadvantage. This bias may also extend to other high-stakes scenarios that rely on remote AV connections; for example, it is possible that judgments made in virtual courts are more favorable to the defendant when AV quality is better (Terry, Johnson, & Thompson, 2010).

If HR professionals and other interviewers want to find a way to diminish the AV quality bias, it appears that they will need to do more than simply be aware of the problem. A better solution, long advocated by industrial and organizational psychologists, might be to do fewer interviews. Analytical methods such as pencil-and-paper assessments (Highhouse, 2008) have been shown to be more predictive of job success than unstructured interviews (Vinchur, Schippmann, Switzer III, & Roth, 1998). Even so, employers still value unstructured interviews (Vinchur et al., 1998) and the convenience and cost-effectiveness of video interviews (Chapman & Webster, 2003) will probably ensure their continued use. Future work should therefore continue to investigate potential interventions that offset the AV quality bias.

Future work should also investigate the extent to which AV fluency remains influential in the context of other information. It is an open question how much impact AV fluency would have if participants had access to candidates’ resumes, letters of recommendation, and so forth, as they would in a real-life interview.

## Footnotes

1 We ceased data collection even though we had not reached the criterion for stopping stated in our preregistration document, which was 0.33. We were primarily interested in the effect of fluency on employability ratings and so we elected to stop collecting data once we had obtained convincing evidence for that comparison.
